# Myostatin antisense administration prevents sepsis‐induced muscle atrophy and weakness in male mice

**DOI:** 10.14814/phy2.70566

**Published:** 2025-09-12

**Authors:** Nobuto Nakanishi, Kazuhiro Maeta, Yuko Ono, Takumi Hirabayashi, Daisuke Tatebayashi, Kensuke Nakamura, Shigeaki Inoue, Masafumi Matsuo, Joji Kotani

**Affiliations:** ^1^ Division of Disaster and Emergency Medicine, Department of Surgery Related Kobe University Graduate School of Medicine Kobe Hyogo Japan; ^2^ Quality Assurance Section, Pharmaceutical Quality Assurance Dept. KNC Laboratories Co., Ltd. Izumo Shimane Japan; ^3^ Division of Rehabilitation Medicine Kobe University Hospital Kobe Hyogo Japan; ^4^ Division of Rehabilitation Sapporo Medical University Hospital Sapporo Hokkaido Japan; ^5^ Department of Emergency and Critical Care Medicine Wakayama Medical University Wakayama Japan; ^6^ Graduate School of Science, Technology and Innovation Kobe University Kobe Hyogo Japan

**Keywords:** antisense, atrophy, muscle, myostatin, sepsis

## Abstract

Muscle atrophy and weakness are serious problems associated with sepsis. However, only a few pharmacological interventions are available to date. In this study, myostatin antisense was used to prevent sepsis‐induced muscle atrophy and weakness. Sepsis was induced in 8‐week‐old male C57BL/6J mice via intraperitoneal injection of 1 mg/g cecal slurry (CS). Myostatin antisense was injected into the right tibialis anterior muscle. Myostatin mRNA was measured in the tibialis anterior and quadriceps femoris muscles on Day 1. The body weight, grip strength, and cross‐sectional area of the tibialis anterior muscle were measured on Day 6. The administration of myostatin antisense decreased myostatin expression on Day 1 in the injected side (0.023 ± 0.010 in CS vs. 0.008 ± 0.002 in CS + antisense) as well as in the noninjected muscles. It also decreased the myostatin protein level (2.0 ± 0.3 in CS vs. 1.2 ± 0.5 in CS + antisense, *p* = 0.04). Body weight reduction (94.9% ± 2.0% vs. 98.2% ± 1.8%, *p* < 0.01) and grip strength reduction (77.0% ± 12.3%vs. 89.8% ± 8.3%, *p* = 0.04) were suppressed by the injection. The cross‐sectional area of the right tibialis anterior muscle increased after the treatment (1116 ± 530 μm^2^ vs. 1435 ± 648 μm^2^, *p* < 0.01). Myostatin antisense suppressed the elevation of myostatin mRNA expression in whole muscles of mice with sepsis and prevented sepsis‐induced muscle atrophy and weakness.

## 1 INTRODUCTION

The mortality of sepsis has declined over the past decade because of improved management (Hotchkiss et al., 2016; Zimmerman et al., 2013). However, physical impairment remains a serious problem (Jones & Griffiths, 2013; Mostel et al., 2019), and nearly one‐third of sepsis survivors suffer from physical impairments even after critical illness (Yende et al., 2016). This condition is known as post‐intensive care syndrome (PICS) (Hiser et al., 2023; Nakanishi et al., 2021; Rawal et al., 2017). Prolonged physical impairment is due to muscle atrophy induced by sepsis (Nakanishi, Takashima, & Oto, 2020; Yoshihara et al., 2023). Muscle atrophy in the acute phase is primarily due to systemic inflammation (Ji et al., 2022; Webster et al., 2020), and approximately 2% of muscle mass atrophy occurs daily after ICU admission (Fazzini et al., 2023; Nakanishi, Oto, et al., 2020). Although the prevention of muscle atrophy is necessary to prevent PICS (Lad et al., 2020), no clinically useful pharmacological intervention for its prevention has been developed to date (Huang et al., 2022).

Muscle atrophy is due to various cascades, including the ubiquitin proteasome, autophagy, and apoptosis pathways (Sartori et al., 2021; Singh et al., 2021; Xia et al., 2021). One of the most important pathways is the myostatin‐related pathway (Carnac et al., 2007; Lee et al., 2021). Myostatin, known as growth differentiation factor‐8, causes muscle atrophy by activating the activin type II receptor pathway (Baczek et al., 2020; Lee & McPherron, 2001; Rodgers & Ward, 2022). Genetic deficiency of myostatin leads to increased muscle mass in various species, including humans (Coleman et al., 2016; Grobet et al., 1997; Schuelke et al., 2004). Myostatin administration also leads to decreased muscle mass in mice (Zimmers et al., 2002). In septic mice, knocking out the myostatin gene prevents sepsis‐induced muscle atrophy (Kobayashi et al., 2021). Therefore, the pharmacological inhibition of myostatin can prevent sepsis‐induced muscle atrophy.

Recently, Maeta et al. found a novel myostatin‐specific splicing inhibitor (myostatin antisense) that could suppress myostatin production (Maeta et al., 2022; Maeta et al., 2023). Splicing is an important process for creating mRNA from genes (Havens et al., 2013). Myostatin mRNA is produced through splicing, in which introns are excised and exons are bonded to pre‐mRNA (Wongpalee & Sharma, 2014). Myostatin antisense combines the specific exon region and inhibits exon bonding, leading to the inhibition of mRNA maturation. Considering that myostatin antisense inhibits myostatin production, we hypothesized that myostatin antisense administration could prevent sepsis‐induced muscle atrophy and weakness in mice. The aims of this study were: (1) to evaluate the trend of myostatin levels in muscle and blood; (2) to assess the impact of myostatin antisense on myostatin and other molecular markers related to muscle atrophy pathways in the muscles of cecal slurry (CS)‐injected septic mice; and (3) to investigate its effect on muscle atrophy and weakness in septic mice. We anticipate that treatment with myostatin antisense will significantly attenuate muscle atrophy and improve muscle strength, thereby providing a potential therapeutic strategy for post‐sepsis muscle impairment.

## 2 MATERIALS AND METHODS

### 2.1 Ethics

This experiment was approved on July 2021 by the Committee on the Ethics of Animal Experiments of Kobe University Graduate School of Medicine (P210704). All procedures were performed in accordance with the recommendations of the International Expert Consensus Initiative for Improvement of Animal Modeling in Sepsis (Osuchowski et al., 2018). This study is reported in accordance with Animal Research: Reporting of In Vivo Experiments (ARRIVE) guidelines.

### 2.2 Animals

In this study, 7‐week‐old male C57BL/6J mice were obtained from Jackson Laboratory Japan (Yokohama, Japan). The mice were maintained on a 12:12 h light: dark cycle at 22°C. A standard rodent diet (CLEA Rodent Diet CE‐7; Clea, Osaka, Japan) and water were provided ad libitum. All mice were acclimatized for a week before beginning the test. Therefore, we tested 8‐week‐old male C57BL/6J mice. Body weight and grip strength were measured on specific days. Mice were euthanized by cervical dislocation under isoflurane anesthesia (Pfizer Japan Inc., Tokyo, Japan), and the tibialis anterior muscle, quadriceps femoris muscle, and blood samples were collected. Collected organs were frozen in liquid nitrogen and stored at −80°C or used for histology analysis.

### 2.3 Preparation of the cecal slurry

CS was prepared as previously reported (Nakanishi et al., 2022): The cecum from the sacrificed mice was ground through a 70‐μm mesh cell strainer (EASYstrainer™, Greiner Bio‐One, Kremsmünster, Austria). The sample was mixed with 1–2 mL of sterile phosphate‐buffered saline (PBS, Wako, Osaka, Japan) and filtered two times. The mixture was centrifuged at 11,000 rpm for 1 min. The supernatant was removed, and the residue was mixed with 15% glycerol PBS to achieve a concentration of 500 mg/mL. The product (400–500 μL) was stored at −80°C in cryogenic biobanking tubes (Greiner Bio‐one, Kremsmünster, Austria).

### 2.4 Sepsis model by cecal slurry injection

Sepsis model using CS is a well‐established method for polymicrobial sepsis (Cai et al., 2023). CS (1 mg/g body weight) was injected intraperitoneally into 8‐week‐old male mice to induce polymicrobial sepsis (Starr et al., 2014), whereas an equal amount of vehicle (15% glycerol PBS) was injected into the control. CS induction was confirmed by a 10% drop in body weight 1 day after CS injection. In these mice, infection was visually confirmed by bacterial culture of blood, muscle, spleen, and liver (Figure S1).

### 2.5 Real‐time PCR

RNA was extracted from the tibialis anterior muscle or quadriceps femoris muscle by homogenization in a TRIzol reagent (#15596026, Invitrogen, Carlsbad, CA, USA), followed by complementary DNA (cDNA) reverse transcription using a high‐capacity cDNA reverse transcription kit (#4368814, Applied Biosciences, Foster City, CA, USA). Real‐time PCR analyses were performed using the Takara Thermal Cycler Dice Real Time System (Takara Bio Inc., Shiga, Japan). Myostatin was measured by using TaqMan™ primers of Mstn (Mm01254559_m1, Thermo Fisher Scientific, Waltham, MA, USA) and glyceraldehyde 3‐phosphate dehydrogenase (GAPDH, Mm99999915_g1, Thermo Fisher Scientific) with TaqMan Fast Advanced Master Mix (4444557, Thermo Fisher Scientific). *FOXO3*, *SMAD2*, *IL‐6*, and *TNF‐α* were measured using KOD SYBR qPCR Mix (#QPS‐201, Toyobo, Osaka, Japan), and the sequences of primers were shown in Table S1. The thermal cycling profile included an initial denaturation at 95°C for 30 s, followed by combined annealing/extension at 60°C for 30 s. This was followed by 40 cycles of denaturation at 95°C for 5 s and annealing/extension at 60°C for 30 s, according to the manufacturer's protocol for the respective qPCR reagents. GAPDH was used as a control to normalize mRNA expression in arbitrary units, and the relative expression was analyzed using the double‐delta cycle threshold (ΔΔCT) method.

### 2.6 Enzyme‐linked immunosorbent assay

Serum myostatin levels were measured using an ELISA kit (DGDF80, R&D System, Minneapolis, MN, USA). In ELISA, all samples were tested in duplicate following the manufacturer's instructions, and the average value was used for analysis. A standard curve was generated by plotting the absorbance and concentration values of the standards using a microplate reader (#168‐1130JP, iMark™ Microplate Reader, Bio‐Rad, Hercules, CA, USA).

### 2.7 Myostatin antisense

We used a recently reported myostatin‐specific splicing inhibitor termed myostatin antisense (KNC Laboratories Co., Ltd., Kobe, Japan) (Maeta et al., 2022; Maeta et al., 2023). This myostatin antisense comprises an 18‐mer chimeric antisense oligonucleotide complementary to the splicing enhancer sequence within exon 1 of the myostatin gene. Myostatin antisense silences gene function through antisense oligonucleotide‐mediated mRNA expression reduction. This antisense is a chimeric antisense oligonucleotide comprising 2′‐OMe RNA and ENA, which is characterized by high affinity for complementary RNA and high stability. A 20‐μL myostatin antisense was injected into the right tibialis anterior muscle immediately after CS injection, whereas 20 μL of PBS was used for comparison. In confirming the concentration‐dependent effect, 5, 10, 20, and 40 μg of myostatin antisense were injected to CS‐injected mice with the same amount to control mice (15% glycerol‐injected mice). Myostatin antisense was injected immediately after intraperitoneal injection of CS or 15% glycerol PBS. After the determination of myostatin antisense injection dose, mice were classified into three groups: control (peritoneal injection of 15% glycerol PBS [1 mg/g bodyweight]), CS (peritoneal injection of CS [1 mg/g bodyweight] and intramuscular injection of 20 μL of PBS to the anterior tibialis muscle), and CS + antisense (peritoneal injection of CS [1 mg/g bodyweight] and intramuscular injection of myostatin antisense [5 μg/20 μL of PBS] to the right tibialis anterior muscle). Blood tests, except for myostatin, were conducted in Oriental Yeast Company (Tokyo, Japan). Tibia length was measured to normalize tibialis anterior muscle weight. Mortality was assessed over a week in CS‐injected mice, CS‐injected mice with myostatin antisense, and control (15% glycerol‐injected mice with PBS intramuscular injection). The overall study plan was presented in Table S2.

### 2.8 Western blot analysis

Western blotting was conducted as reported previously (Ono et al., 2022). Muscle tissues were homogenized and lysed in RIPA lysis buffer. Equal amounts of protein (10–20 μg) were applied on 10% or 15% polyacrylamide gels with a protein ladder (ab116028, Abcam, Cambridge, UK). The gel was transferred overnight to polyvinylidene difluoride membranes (Immobilon‐P; Merck Millipore, Darmstadt, Germany) using an XCell SureLock System (Thermo Fisher Scientific). Membranes were blocked with 5% (wt/vol) non‐fat dried milk for 60 min at room temperature and mixed with primary antibodies specific for Myostatin (rabbit monoclonal, ab203076, Abcam, Cambridge, UK; 1:1000 dilution), MyoD (mouse monoclonal, sc‐32758, Santa Cruz Biotechnology, TX, USA; 1:200 dilution), or MuRF1 (mouse monoclonal, sc‐398608, Santa Cruz Biotechnology, TX, USA; 1:500 dilution). The membranes were washed with Tris‐buffered saline containing Tween® 20 (0.1% vol/vol, Sigma Aldrich) three times for 15 min each and mixed with the corresponding horseradish peroxidase‐conjugated secondary antibody (1:3000 dilution of goat anti‐mouse IgG, 62‐6520 or goat anti‐rabbit IgG, 65‐6120, Thermo Fisher Scientific). The protein bands image was captured using Clarity Western ECL Substrate (Clarity™ Western ECL Substrate, 1705060, Bio‐Rad, CA, USA) and Amersham Imager 600 image analysis software (GE Healthcare Life Sciences, Piscataway, NJ, USA). The density of the visualized bands was quantified using ImageJ. Data were shown as the normalized ratio of protein band density against β‐tubulin (rabbit polyclonal, ab6046, Abcam, Cambridge, UK; 1:1000 dilution).

### 2.9 Histology

The tibialis anterior muscle was dissected from the right legs at Day 6. Each group included three mice. The dissected tibialis anterior muscle was immersed in 4% paraformaldehyde containing 0.2% picric acid for at least 1 day. The right tibialis anterior muscle was paraffin embedded and sectioned at a thickness of 5 μm. The sections were stained with a hematoxylin and eosin solution (Wako, Osaka, Japan) and examined using a light microscope equipped with a camera (Olympus BX43, Tokyo, Japan). For the quantification of the myofiber cross‐sectional area, ImageJ was used to capture images at 20× magnification (National Institutes of Health, Bethesda, MD, USA). In calculating the myofiber cross‐sectional area, approximately 200 myofibers were measured in at least four fields of view per group.

### 2.10 Grip strength

Grip strength was measured using a grip strength meter (MK‐380Si; Muromachi Kikai, Tokyo, Japan) 6 days after CS injection in the three abovementioned groups. The tail was pulled back, and the mice used their front paws to grab the bar of the grip strength meter. The grip strength was measured three times, and the highest value was used for evaluation as previously described (Ono et al., 2020; Ono et al., 2022).

### 2.11 Statistical analysis

Data distribution was assessed using the Shapiro–Wilk test, and since most data were normally distributed, statistical analyses were standardized using parametric tests. Continuous data were expressed as the mean ± standard deviation and compared using the *t*‐test or one‐way analysis of variance (ANOVA) for repeated measures. Post hoc correction for multiple comparisons was performed with Dunnett or Tukey's test. All statistical tests were two‐tailed, and statistical significance was defined as a *p*‐value of 0.05. JMP statistical software version 13.1.0 (SAS Institute Inc., Cary, NC, USA) was used for statistical analysis.

## 3 RESULTS

### 3.1 Myostatin in the tibialis anterior muscle and blood

**FIGURE 1:**
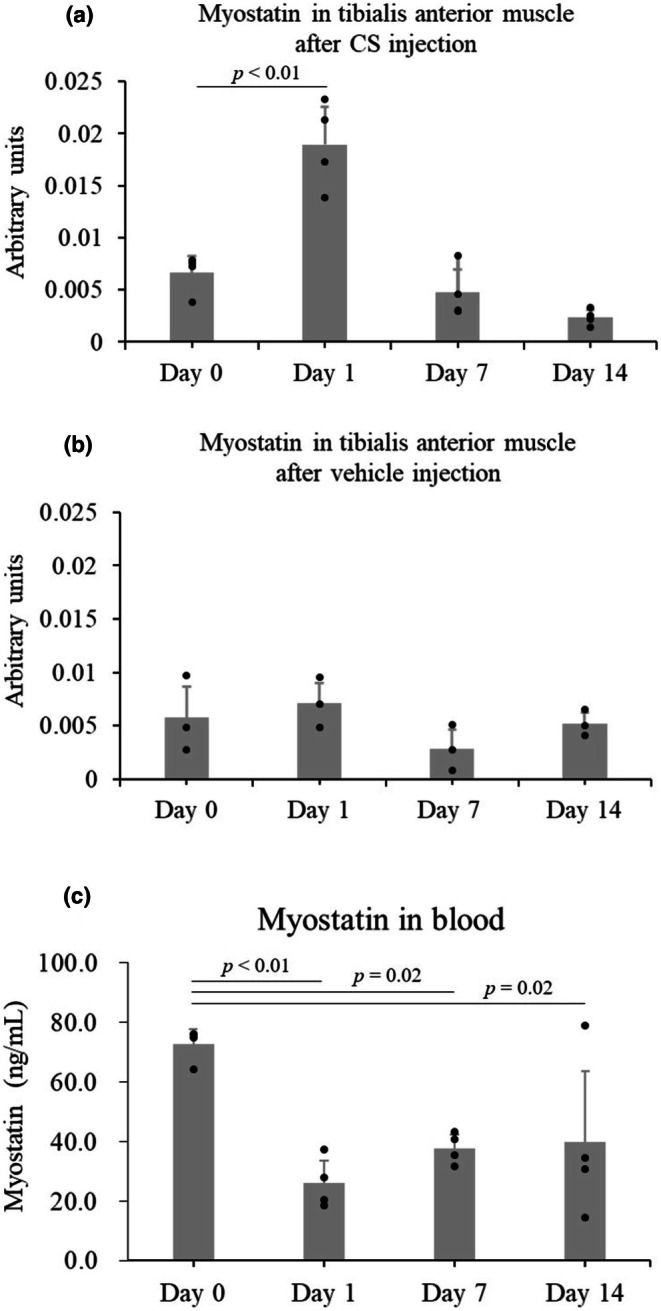
Longitudinal changes in the relative expression of myostatin mRNA (arbitrary units) in the tibialis anterior muscle after CS injection, tibialis anterior muscle after vehicle injection, and blood after CS injection. (a) Relative expression of myostatin mRNA in the right tibialis anterior muscle after CS injection, (b) Relative expression of myostatin mRNA in the right tibialis anterior muscle after vehicle injection (15% glycerol PBS), and (c) Myostatin concentration in the blood measured by ELISA after CS injection. Each group included three mice in (b) or four in (a and c). Data are shown as the mean ± standard deviation and compared with day 0 using the one‐way analysis of variance (ANOVA) for repeated measures with post hoc Dunnett test. CS, cecal slurry; ELISA, enzyme‐linked immunosorbent assay.

After CS injection, defined as Day 0, the longitudinal change in myostatin expression in the tibialis anterior muscle and blood was investigated. Each study day included four mice. In the tibialis anterior muscle, myostatin expression increased 1 day after CS injection (0.007 ± 0.002 in day 0 vs. 0.019 ± 0.004 in Day 1, *p* < 0.01) and decreased thereafter (0.005 ± 0.002 in Day 7, *p* = 0.63; 0.002 ± 0.001 in Day 14, *p* = 0.10, Figure 1a), whereas there were no significant changes after vehicle (15% glycerol) injection (Figure 1b). In the blood, myostatin levels decreased 1 day after CS injection (72.8 ± 4.9 ng/mL in Day 0 vs. 26.1 ± 7.4 ng/mL in Day 1, *p* < 0.01) and slightly increased thereafter (37.8 ± 4.5 ng/mL in Day 7, *p* = 0.02; 39.7 ± 23.9 ng/mL in Day 14, *p* = 0.02, Figure 1c).

### 3.2 Myostatin expression in various muscles after myostatin antisense injection

**FIGURE 2:**
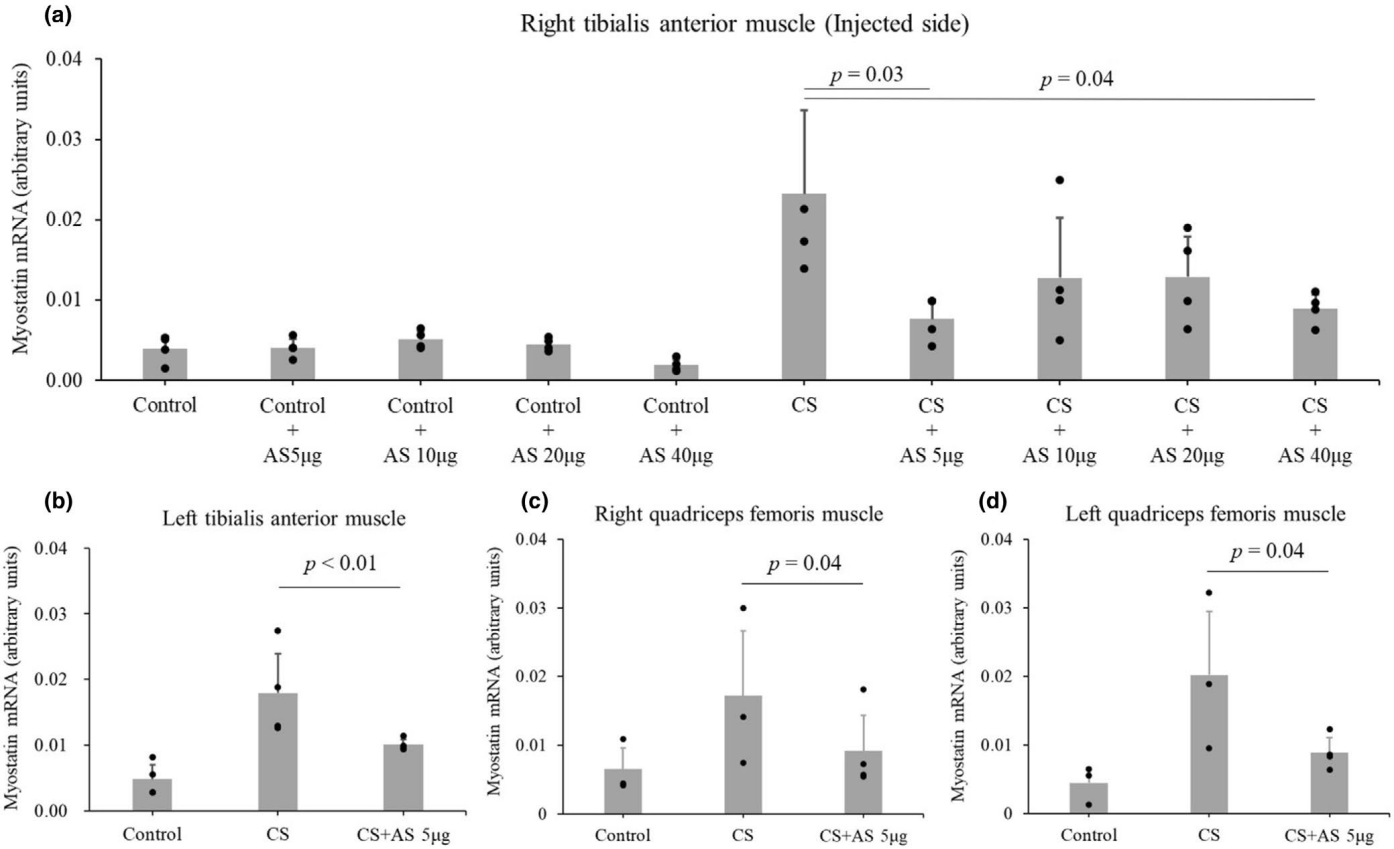
Effect of myostatin antisense in various muscles. Different doses of myostatin antisense were injected into the right tibialis anterior muscle of CS‐injected mice and vehicle‐injected mice (control). One day after CS injection, the myostatin mRNA expression relative to GAPDH mRNA (arbitrary units) was examined in (a) right tibialis anterior muscles. After confirming a non‐dose‐dependent effect, the effect of 5 μg of myostatin antisense was investigated in different muscles of CS‐injected mice and vehicle‐injected mice (control). (b) Left tibialis anterior muscle, (c) Right quadriceps femoris muscle, and (d) Left quadriceps femoris muscle. Each group included four mice in (a and b) or three mice in (c and d). Data are shown as the mean ± standard deviation. The effect of Myostatin antisense injection (CS + AS) was compared with CS using one‐way analysis of variance (ANOVA) for repeated measures with a post hoc Dunnett test in the right tibialis anterior muscle and Tukey's test for the remaining muscles. AS, antisense; CS, cecal slurry.

Dose‐dependent effect of myostatin antisense was investigated in CS and control mice. Each group included four mice. One day after CS and myostatin antisense injection, myostatin expression decreased in the tibialis anterior muscle of the myostatin antisense–injected side (0.023 ± 0.010 in CS vs. 0.008 ± 0.002 in CS + antisense 5 μg, *p* = 0.03, Figure 2a). The myostatin expression did not decrease in a dose‐dependent manner (0.013 ± 0.007 in CS + antisense 10 μg, *p* = 0.17; 0.013 ± 0.005 in CS + antisense 20 μg, *p* = 0.17; 0.009 ± 0.002 in CS + antisense 40 μg, *p* = 0.04). Myostatin antisense injection did not change the myostatin expression in control mice (15% glycerol‐injected mice). The effect of myostatin antisense was evaluated in non‐injected muscles. Each group included four mice. Under 5 μg of myostatin antisense, myostatin expression decreased in the left tibialis anterior muscle (0.018 ± 0.006 in CS vs. 0.010 ± 0.001 in CS + antisense, *p* < 0.01, Figure 2b), right quadriceps femoris muscle (0.017 ± 0.010 in CS vs. 0.009 ± 0.005 in CS + antisense, *p* = 0.04, Figure 2c), and left quadriceps femoris muscle (0.020 ± 0.009 in CS vs. 0.009 ± 0.002 pg/mL in CS + antisense, *p* = 0.04, Figure 2d).

### 3.3 Real‐time PCR regarding the effect of myostatin antisense

**FIGURE 3:**
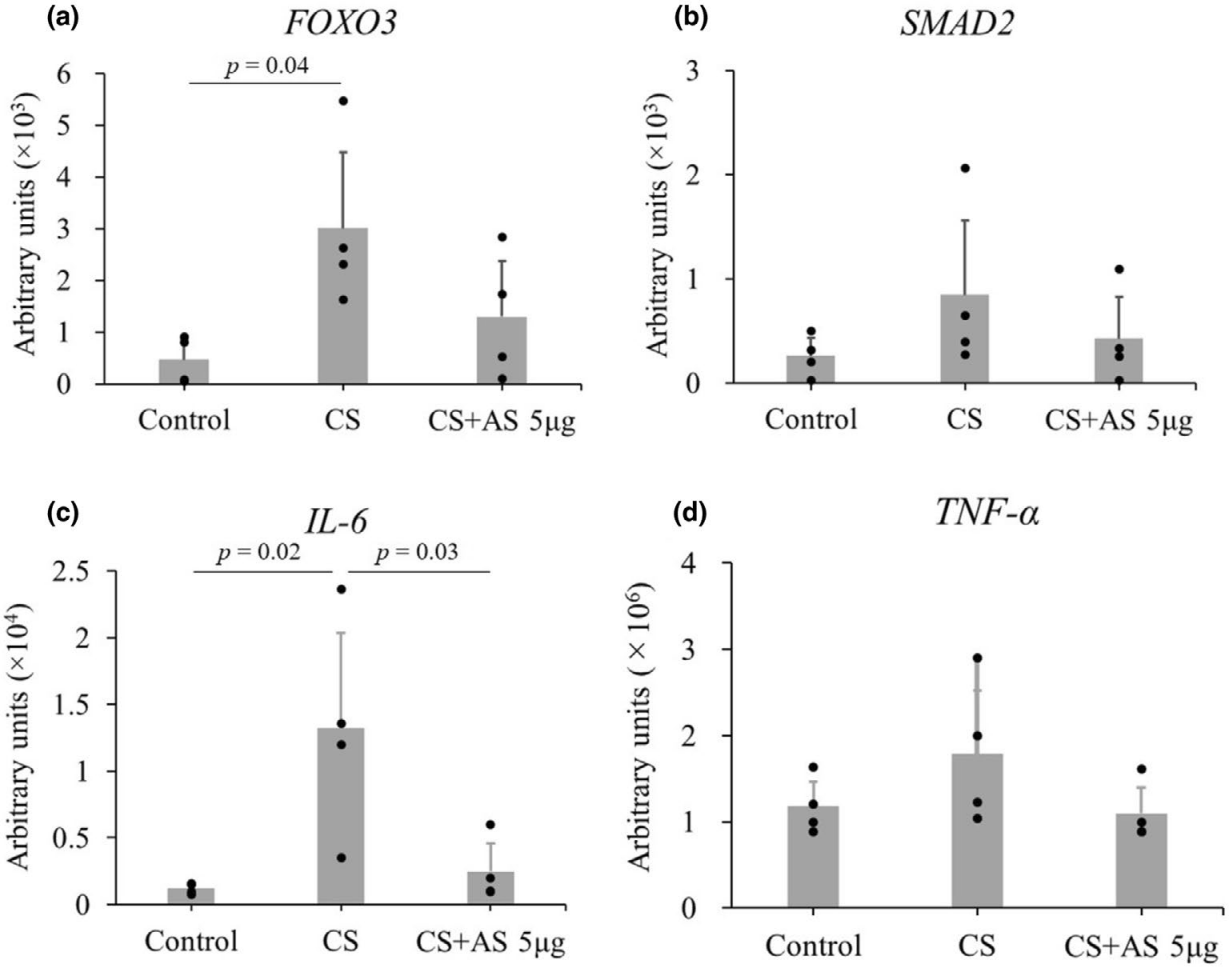
Real‐time PCR regarding the effect of myostatin antisense. The effect of myostatin antisense on mRNA relative expression was compared among control, CS, and CS with myostatin antisense 1 day after its injection. (a) *FOXO3*, (b) *SMAD2*, (c) *IL‐6*, (d) *TNF‐α*. Each group included four mice. Data are shown as the mean ± standard deviation. The effect of Myostatin antisense injection (CS + AS) was compared with control or CS using one‐way analysis of variance (ANOVA) for repeated measures and post hoc Tukey's test. AS, antisense; CS, cecal slurry.

In right tibialis anterior muscle, myostatin antisense slightly decreased the mRNA relative expression of *FOXO3* without statistical significance (0.00301 ± 0.00147 in CS vs. 0.00130 ± 0.00107 in CS + AS, *p* = 0.18, Figure 3a) and *SMAD2* (0.000846 ± 0.000716 in CS vs. 0.000425 ± 0.000400 in CS + AS, *p* = 0.56, Figure 3b). Regarding inflammatory cytokine levels in right tibialis anterior muscle, the mRNA relative expression of *IL‐6* and *TNF‐α* decreased 1 day after 5 μg of myostatin antisense injection. In right tibialis anterior muscle, myostatin antisense decreased the mRNA relative expression of *IL‐6* (0.00000132 ± 0.00000072 in CS vs. 0.00000025 ± 0.00000021 in CS + AS, *p* = 0.03, Figure 3c) and *TNF‐α* (0.00000179 ± 0.00000073 in CS vs. 0.00000110 ± 0.00000030 in CS + AS, *p* = 0.24, Figure 3d).

### 3.4 Western blot analysis regarding the effect of myostatin antisense

**FIGURE 4:**
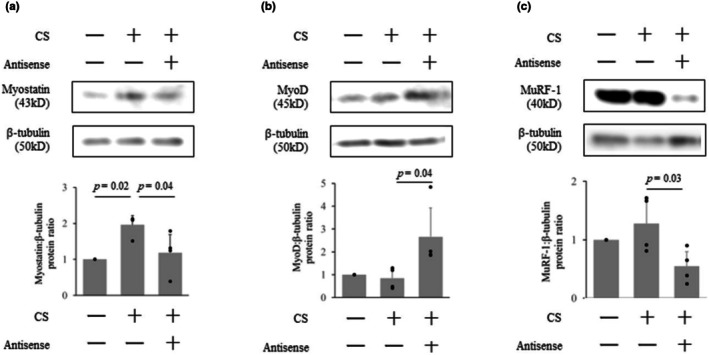
Western blot analysis regarding the effect of myostatin antisense. The effect of myostatin antisense on protein level was compared among control, CS, and CS with myostatin antisense 2 days after its injection. (a) Myostatin, (b) MyoD, (c) MuRF‐1. The density of the visualized bands was quantified, and data were shown as the normalized ratio of protein band density against β‐tubulin. Each group included four mice. The original image of western blot analysis was shown in Figure S3. Data are shown as the mean ± standard deviation. The effect of Myostatin antisense injection (CS + AS) was compared with control or CS using one‐way analysis of variance (ANOVA) for repeated measures and post hoc Tukey's test. AS, antisense; CS, cecal slurry.

The effect of myostatin antisense on protein level was compared among control, CS, and CS with myostatin antisense 2 days after its injection. Each group included four mice. The myostatin protein level significantly decreased by myostatin antisense injection (2.0 ± 0.3 in CS vs. 1.2 ± 0.5 in CS + antisense, *p* = 0.04, Figure 4a). The MyoD level significantly increased by myostatin antisense injection (0.9 ± 0.4 in CS vs. 2.7 ± 1.3 in CS + antisense, p = 0.04, Figure 4b). On the other hand, MuRF‐1 level significantly decreased (1.3 ± 0.4 in CS vs. 0.6 ± 0.3 in CS + antisense, *p* = 0.03, Figure 4c) by myostatin antisense injection.

### 3.5 Effect of myostatin antisense 6 days after CS and antisense injection

**FIGURE 5:**
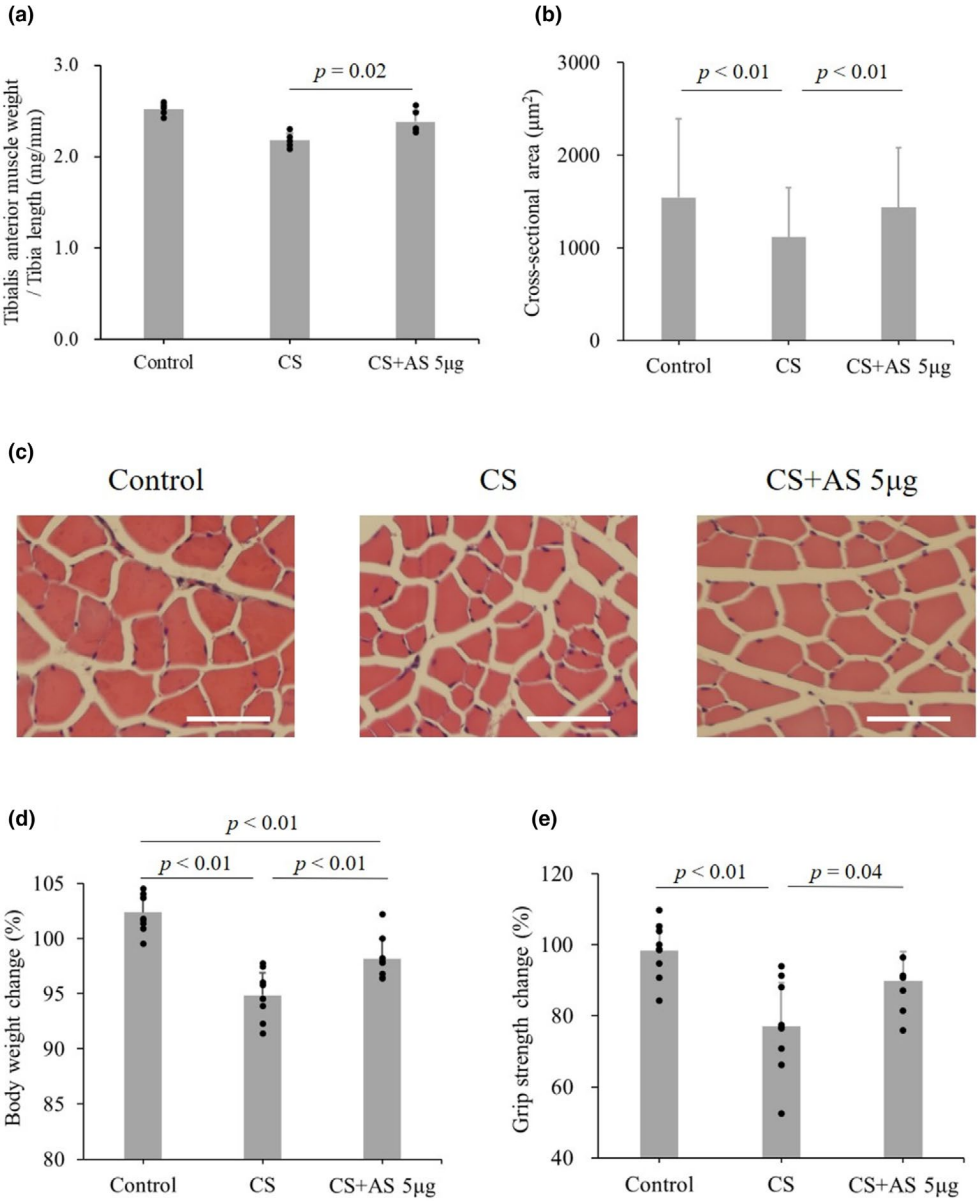
Effect of myostatin antisense 6 days after CS and antisense injection. The effect of myostatin antisense was compared among the control, CS, and CS + AS 6 days after its administration. (a) Right tibialis anterior muscle weight normalized to tibia length, (b) Right tibialis anterior muscle cross‐sectional area, (c) Histology of the right tibialis anterior muscle cross‐sectional area, scale bar is 100 μm. (d) Body weight change, and (e) Grip strength change. Each group included five (a), three (b), and nine mice (d, e). Data are shown as the mean ± standard deviation. The effect of Myostatin antisense injection (CS + AS) was compared with control and CS using one‐way analysis of variance (ANOVA) for repeated measures and post hoc Tukey's test. AS, antisense; CS, cecal slurry.

The effect of myostatin antisense was compared among control, CS, and CS with myostatin antisense 6 days after its injection. There was no statistically significant difference in general blood tests (Table S3) and survival rate (Figure S2) as well as myostatin in blood (47.4 ± 15.8 ng/mL in Control, 48.8 ± 20.3 ng/mL in CS, 37.9 ± 27.0 ng/mL in CS + antisense, *p* = 0.74, 5 mice in each group). The right tibialis anterior muscle weight normalized by tibia length significantly increased by myostatin antisense injection (2.2 ± 0.1 in CS vs. 2.4 ± 0.1 in CS + antisense, *p* = 0.02, five mice in each group, Figure 5a). On the contrary, the cross‐sectional area of the right tibialis anterior muscle was increased in CS mice injected with myostatin antisense (1116 ± 530 μm^2^ in CS vs. 1435 ± 648 μm^2^ in CS + antisense, *p* < 0.01, three mice in each group, Figure 5b). The histological difference was visually shown in Figure 5c. Body weight (94.9% ± 2.0% in CS vs. 98.2% ± 1.8% in CS + antisense, *p* < 0.01, nine mice in each group, Figure 5d) and grip strength (77.0% ± 12.3% in CS vs. 89.8% ± 8.3% in CS + antisense, *p* = 0.04, nine mice in each group, Figure 5e) significantly increased by myostatin antisense injection.

## 4 DISCUSSION

In this study, we investigated the effect of the myostatin‐specific splicing inhibitor “myostatin antisense” in a sepsis mouse model to prevent sepsis‐induced muscle atrophy and weakness. We determined the effective dose of myostatin antisense and found that myostatin antisense decreased the rapid elevation of myostatin mRNA expression in various muscles in the whole body. Consequently, myostatin antisense treatment prevented muscle atrophy and grip strength impairments. This result indicates that future pharmacological intervention is promising to prevent sepsis‐induced muscle atrophy and impairments.

In this study, we injected myostatin antisense only into the right tibialis anterior muscle. However, the effect was observed in both quadriceps muscles and the left tibialis anterior muscle. These findings provide supportive evidence for the systemic effects of locally administered myostatin antisense. Myostatin antisense reduces mature mRNA expression in pre‐mRNA maturation through the steric inhibition of splicing enhancers within exon 1. Since FOXO3 and SMAD2 are the downstream cytokines of myostatin, these reductions by myostatin antisense are reasonable. Western blot analysis suggested the interaction of MyoD and MuRF‐1 related pathways. The suppression of myostatin expression is complicatedly connected to various cascades. Among them, SMAD2, MyoD, and embryonic myosin heavy chain are important factors in muscle regeneration (Schiaffino et al., 2015). Previous studies have reported that inhibition of Smad2/3 activation promotes muscle hypertrophy (Nielsen et al., 2021), and the knockout of embryonic myosin heavy chain results in muscle atrophy (Bharadwaj et al., 2023). Myostatin antisense treatment may have influenced muscle regeneration, suggesting a potential avenue for future research. Considering that myostatin works in an autocrine or paracrine manner (Lee et al., 2016), the locally injected myostatin antisense was considered to be distributed throughout the body.

Interestingly, we found that myostatin antisense treatment significantly reduced IL‐6, a proinflammatory cytokine. Inflammation is a major contributor to muscle atrophy and weakness in sepsis (Yoshihara et al., 2023). Therefore, suppression of inflammatory cytokines could potentially help prevent these complications. In particular, the observed reduction in IL‐6 may reflect a broader anti‐inflammatory effect of myostatin inhibition. Previous studies have suggested that myostatin influences not only muscle homeostasis but also immune cell function, potentially modulating host defense and inflammatory responses (Zhou et al., 2010). Thus, suppression of myostatin signaling could indirectly attenuate inflammatory pathways and, in turn, reduce muscle atrophy. These findings are promising for future clinical application. Because locally administered myostatin antisense exerts systemic effects, it holds potential as a clinically viable therapeutic approach.

Antisense treatment uses RNA‐binding antisense oligonucleotides, and it has received increasing attention as a next‐generation medication (Lee & Yokota, 2013). Another study has reported the effect of another myostatin inhibitor “YK11,” which modulates the effect of the selective androgen receptor to inhibit myostatin production (Lee et al., 2021). The myostatin inhibitor YK11 has preventive effects against sepsis‐induced muscle atrophy. Antisense therapy has a different functional mechanism, directly targeting nucleic acids (Lee & Yokota, 2013; Roberts et al., 2020). The benefit of this myostatin antisense is the high specificity for myostatin. Myostatin and GDF‐11 are closely related TGF‐β family members, and the lack of specific function on myostatin may cause unwanted side effects (Long et al., 2018). Due to the high homology between myostatin and GDF‐11, specific inhibition of myostatin is difficult. However, the specificity was observed in a previous study using this myostatin antisense (Maeta et al., 2022).

Unlike previous research, our study did not find any significant difference in biomarkers or mortality. Lee et al. reported that the myostatin inhibitor YK11 decreased organ damage biomarkers and mortality (Lee et al., 2021). Our results supported this finding, but did not reach statistical significance. The sepsis mouse model and its severity differed from the previous study. We used a CS model, in which the severity depends on the amount of cecal slurry injected. We used a dose of 1 mg/g of CS to investigate muscle atrophy and function, not mortality. Consequently, mortality was limited to 10%, which may explain the lack of statistical significance. In the previous study, they used E. coli K1 strain, and the mortality reached 100% within 32 h, while YK 11 extended survival to around 72 h. This result suggests that our CS model was not suitable for investigating mortality. The same reasoning may apply to blood biomarkers. In our CS model, creatinine levels increased from Control: 0.10 ± 0.01 mg/dL to CS model: 0.13 ± 0.01 mg/dL, and decreased to CS + Antisense: 0.12 ± 0.01 mg/dL (*p* = 0.07). A more severe sepsis model may reveal the organ‐protective effects and improved survival with a statistical difference.

There was no dose‐dependent effect of myostatin antisense, and 5 μg dose may be sufficient to decrease myostatin level in muscle. The lack of dose‐dependence may suggest that the myostatin antisense regulation of myostatin expression becomes saturated at lower doses when administered as a single injection. In this study, we used a one‐time injection of myostatin antisense immediately after sepsis induction. The number of doses tested, rather than a bolus injection, could potentially influence the dose‐dependent results. Insufficient intervals between doses might obscure a true dose‐dependent relationship. It could be useful to explore whether myostatin inhibition has a threshold effect, beyond which increasing the frequency of injection reduces myostatin expression. Another reason may relate to the sepsis mouse model. We used a CS mouse model with a 1.0 mg/g CS dose. In a more severe sepsis model, a dose‐dependent effect might be observed, which warrants further investigation.

In a human study, serum myostatin levels were lower in critically ill patients than in healthy volunteers, and lower myostatin levels were associated with increased mortality (Wirtz et al., 2020). Wirtz et al. investigated serum myostatin levels in 165 critically ill patients and found that low myostatin levels were an independent prognostic marker for survival (hazard ratio: 0.433, 95% CI: 0.211–0.889). Consistent with this finding, in our study, the serum myostatin level in septic mice decreased, but myostatin expression increased within the muscles. The mechanism can be explained by the autocrine or paracrine manner of myostatin excretion. Myostatin is primarily produced from skeletal muscle, and it acts locally in an autocrine or paracrine manner (Lee et al., 2016). Therefore, after sepsis induction, myostatin was consumed in the muscle, which caused the decrease of the serum myostatin level. Another possible mechanism is the myostatin negative feedback inhibition. Increased myostatin in muscle may have suppressed myostatin production and circulation in blood. Additionally, increased sequestration of myostatin by inhibitory proteins such as propeptide, follistatin, or growth and differentiation factor–associated serum protein‐1 could further reduce the measurable circulating fraction, even in the presence of elevated muscle myostatin content.

Another study has reported that myostatin expression decreased in muscle (Grunow et al., 2022). Grunow et al. investigated myostatin gene expression in skeletal muscle among critically ill patients and found that the expression of the muscular myostatin gene was downregulated during the progression of the critical illness. However, this study investigated the muscle 14 days after ICU admission. In our study, myostatin expression in the muscle peaked 1 day after CS injection and decreased thereafter. Consistent with this result, a previous basic study has reported that myostatin mRNA expression in the muscle of mice increased 1 day after sepsis induction (Morel et al., 2017). These results indicate that myostatin expression in muscles rapidly increases in critically ill patients, and the suppression of this rapid increase is the key factor influencing muscle atrophy and weakness.

In this study, we focused only on the acute phase. We aimed to investigate the effect of myostatin antisense administration on PICS. Considering that intervention in the acute phase is important for long‐term outcomes, myostatin antisense intervention will be an important strategy to prevent PICS. Aerde et al. reported that physical impairment acquired during ICU stay was associated with decreased physical function and muscle strength as well as increased mortality even 5 years after discharge (Van Aerde et al., 2020). In another study, muscle loss in the acute phase was associated with prolonged fatigue and myalgia 6 months after discharge (Gil et al., 2023). A meta‐analysis reported that rehabilitation intervention was insufficient to prevent PICS (Fuke et al., 2018). Therefore, the combination of this novel myostatin antisense treatment and conventional care will contribute to the prevention of PICS and subsequent reintegration into society among sepsis survivors.

This study has several limitations. First, this study utilized only male mice, which may limit the generalizability of the findings given known sex differences in immune responses and muscle metabolism (Escrivà‐Font et al., 2025). Second, we focused on the feasibility and effect of myostatin antisense in young mice, and the impact in aged mice remains unknown. Given that aging is associated with altered immune function and muscle regenerative capacity (Tidball et al., 2021), future studies should examine older mice. Third, this study focused primarily on skeletal muscle. However, myostatin is known to be expressed and to exert regulatory effects in various other organs (Luo et al., 2019), including the brain (Han et al., 2023). These extra‐muscular actions may contribute to the systemic manifestations of sepsis and influence overall outcomes. Fourth, this study employed a CS‐injected sepsis model of relatively mild severity, as reflected by modest bacterial burden, limited changes in blood parameters, and low mortality. While this approach facilitated the evaluation of early pathophysiological changes, the mild phenotype may limit the direct translational applicability of our findings to patients with more severe sepsis. Fifth, we did not use non‐targeting AS‐constructs as in our previous study (Maeta et al., 2022). The lack of a non‐targeting control oligonucleotide group limits the ability to fully exclude off‐target or sequence‐independent effects of the oligonucleotide intervention. Including such controls in future experiments will strengthen the specificity of the observed effects and enhance the validity of mechanistic conclusions. Sixth, our investigation focused primarily on the acute phase post‐sepsis induction. However, muscle atrophy and systemic alterations often evolve over a prolonged time course. Longitudinal studies are needed to elucidate the chronic and recovery phases, which will provide a more comprehensive understanding of the disease process and the potential therapeutic window.

## 5 CONCLUSION

The intramuscular administration of myostatin antisense suppressed the rapid elevation of myostatin mRNA expression in whole muscles of mice with sepsis and prevented sepsis‐induced muscle atrophy and weakness. However, future studies must be conducted for its clinical application.

## AUTHOR CONTRIBUTIONS

Nobuto Nakanishi, Shigeaki Inoue, and Yuko Ono took part in the study concept. Nobuto Nakanishi, Takumi Hirabayashi, and Daisuke Tatebayashi carried out the experiments. Nobuto Nakanishi and Kazuhiro Maeta obtained research funding. Shigeaki Inoue, Masafumi Matsuo, and Joji Kotani supervised the project. Nobuto Nakanishi drafted the manuscript, and all authors contributed to the revision. All authors read and approved the final manuscript.

## FUNDING INFORMATION

Kazuhiro Maeta is employed by KNC Laboratories Co., Ltd., Kobe, Japan. This study was supported by JSPS KAKENHI Grant Numbers JP24K19491.

## ETHICS STATEMENT

Ethics approval was obtained from the Committee on the Ethics of Animal Experiments of Kobe University Graduate School of Medicine (P210704).

## Supporting information


Data S1.

## Data Availability

Data are provided within the manuscript and available upon reasonable request from the corresponding author.
