# Supplementary material for: Myostatin antisense administration prevents sepsis‐induced muscle atrophy and weakness in male mice
Source: Physiol Rep. 2025 Sep 12;13(17):e70566. doi: 10.14814/phy2.70566 (PMC12431578; doi:10.14814/phy2.70566)
Supplement: Supplementary file 1 — Data S1. [file PHY2-13-e70566-s001.docx]

Table S1. Primer list for Real Time PCR

| Gene | Primer | Sequence |
| --- | --- | --- |
| *FOXO3* | Forward | 5'-AGGATAAGGGCGACAGCAAC-3' |
|  | Reverse | 5'-CATTCTGAACGCGCATGAAG-3' |
| *SMAD2* | Forward | 5'-AGGCTCCTCATCCCATTCCT-3' |
|  | Reverse | 5'-TGACAAGACCTCAGCGTCAC-3' |
| *IL-6* | Forward | 5'-CCTCTGGTCTTCTGGAGTACC-3' |
|  | Reverse | 5'-ATTGGACGACCACACACACTGCAAG-3' |
| *TNF-α* | Forward | 5'-ACACTCCCAGACCCGGTAT-3' |
|  | Reverse | 5'-CTACCCAACATGGAACAGATGAGG-3' |
| *GAPDH* | Forward | 5'-TGTGTCCCTCGTGGATCTGA-3' |
|  | Reverse | 5'-TTGCTGTTGAAGTCGCAGGAG-3' |

Bacterial culture from tissues

1. Euthanasia of mice
2. Blood, muscle, spleen, liver sampling
3. Measurements of organ weight for homogenate
4. Tissues were filtered through a 40 μm mesh (EASYstrainer™, Greiner Bio-One, Kremsmünster, Austria) into 10 cm dish.
5. The homogenate solution was poured into 50 mL tube through another 40 μm mesh.
6. The homogenate solution was centrifuged at 1500 rpm for 5 minutes.
7. The supernatant was collected, and diluted ten to one thousand times by PBS.
8. The 100 μL of blood or supernatant was dispersed on blood agar medium, and spread by bacteria spreader.
9. The blood agar medium was incubated for 24 hours.


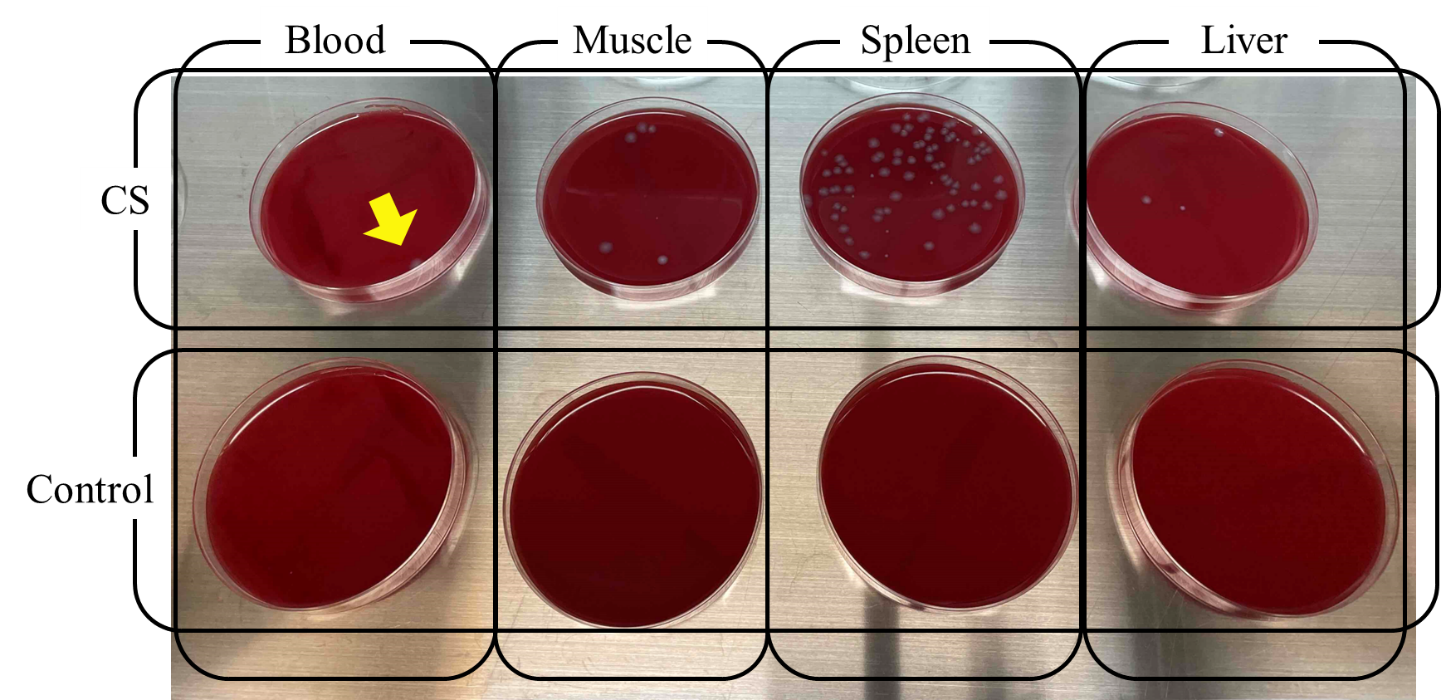


Figure S1. Bacterial culture from blood, muscle, spleen, and liver

The blood agar medium was incubated for 24 hours. Bacterial culture in CS model, blood, muscle, spleen, and liver had one (yellow allow), five, around eighty, three colonies, respectively, whereas bacterial culture in control did not have any colony.

|  | Day 0 | Day 1 | Day 2 | Day 6 |
| --- | --- | --- | --- | --- |
| Event | CS induction  Antisense | Sacrifice | Sacrifice | Sacrifice |
| Body weight |  | ○ |  | ○ |
| Grip strength |  |  |  | ○ |
| Blood test |  |  |  | ○ |
| Real-time PCR |  | ○ |  |  |
| Western blot |  |  | ○ |  |
| Histology |  |  |  | ○ |
| Tibialis anterior muscle  /Tibia length |  |  |  | ○ |

Table S2.

This table shows the overall study plan to evaluate myostatin antisense injection in CS-injected mouse. Since myostatin mRNA rapidly increases after CS injection, real-time PCR was conducted at Day 1. Myostatin protein was measured at the Day 2 because protein is produced after mRNA expression. Finally, all other outcomes were evaluated at Day 6 to assess the effect of myostatin antisense in CS-injected mouse.

| Blood tests | Control | CS | CS + Antisense | p-value |
| --- | --- | --- | --- | --- |
| TP, g/dL  (total protein) | 3.9 ± 0.5 | 3.7 ± 0.2 | 4.3 ± 0.2 | 0.26 |
| ALB, g/dL  (albumin) | 2.7 ± 0.4 | 2.1 ± 0.2 | 2.5 ± 0.2 | 0.14 |
| BUN, mg/dL  (blood urea nitrogen) | 23.3 ± 2.1 | 23.6 ± 3.1 | 24.1 ± 2.9 | 0.96 |
| CRE, mg/dL  (creatinine) | 0.10 ± 0.01 | 0.13 ± 0.01 | 0.12 ± 0.01 | 0.07 |
| Na, mEq/L  (sodium) | 156.3 ± 1.2 | 155.0 ± 0.8 | 154.0 ± 2.2 | 0.37 |
| K, mEq/L  (potassium) | 4.5 ± 0.6 | 4.9 ± 0.2 | 4.9 ± 0.2 | 0.58 |
| Cl, mEq/L  (chlorine) | 116.0 ± 2.9 | 117.7 ± 1.7 | 115.0 ± 1.4 | 0.49 |
| Ca, mg/dL  (calcium) | 7.5 ± 0.9 | 7.6 ± 1.0 | 8.5 ± 0.4 | 0.43 |
| IP, mg/dL  (inorganic phosphorus) | 13.2 ± 1.5 | 12.1 ± 0.9 | 11.8 ± 1.6 | 0.59 |
| AST, IU/L  (aspartate aminotransferase) | 30.3 ± 4.0 | 42.0 ± 11.4 | 47.7 ± 1.7 | 0.12 |
| LDH, IU/L  (lactate dehydrogenase) | 90.7 ± 6.2^A, B^ | 210.7 ± 26.2^A^ | 241.7 ± 15.6^B^ | < 0.01 |
| AMY, IU/L  (amylase) | 1646 ± 268 | 1518 ± 103 | 1925 ± 187 | 0.19 |
| γ-GTP, IU/L  (γ-glutamyl transpeptidase) | 4.0 ± 1.4 | 4.0 ± 1.4 | 3.0 ± 0.0 | 0.63 |
| T-CHO, mg/dL  (total cholesterol) | 71.7 ±11.6 | 53.0 ± 5.0 | 68.3 ± 3.3 | 0.10 |
| TG, mg/dL  (triglyceride) | 42.0 ± 8.5 | 48.7 ± 10.5 | 62.3 ± 6.1 | 0.13 |

Table S3. Blood tests

Several blood tests were conducted in control, CS, and CS + Antisense. Each group included three mice at the total of nine mice. Data were analyzed using analysis of variance (ANOVA) followed by the post hoc Tukey's test. There was a significant difference only in LDH. LDH increased in CS and CS + Antisense, compared with control.

^A, B^ Significant at p < 0.01 (post hoc Tukey's test)

CS: cecal slurry


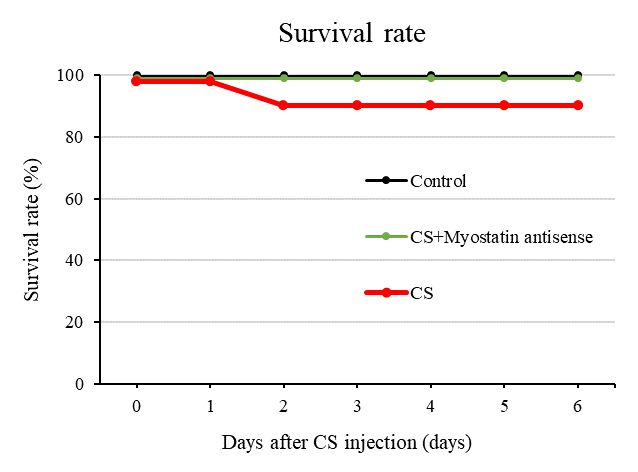


Figure S2. Survival rate of Control, CS, and CS + Myostatin antisense

Survival rate was compared among Control, CS, and CS + Myostatin antisense. Each group included ten mice. One mouse died 2 days after CS injection, whereas no mouse died in Control and CS + Myostatin antisense. There was no overall statistical difference among groups (p = 0.37).


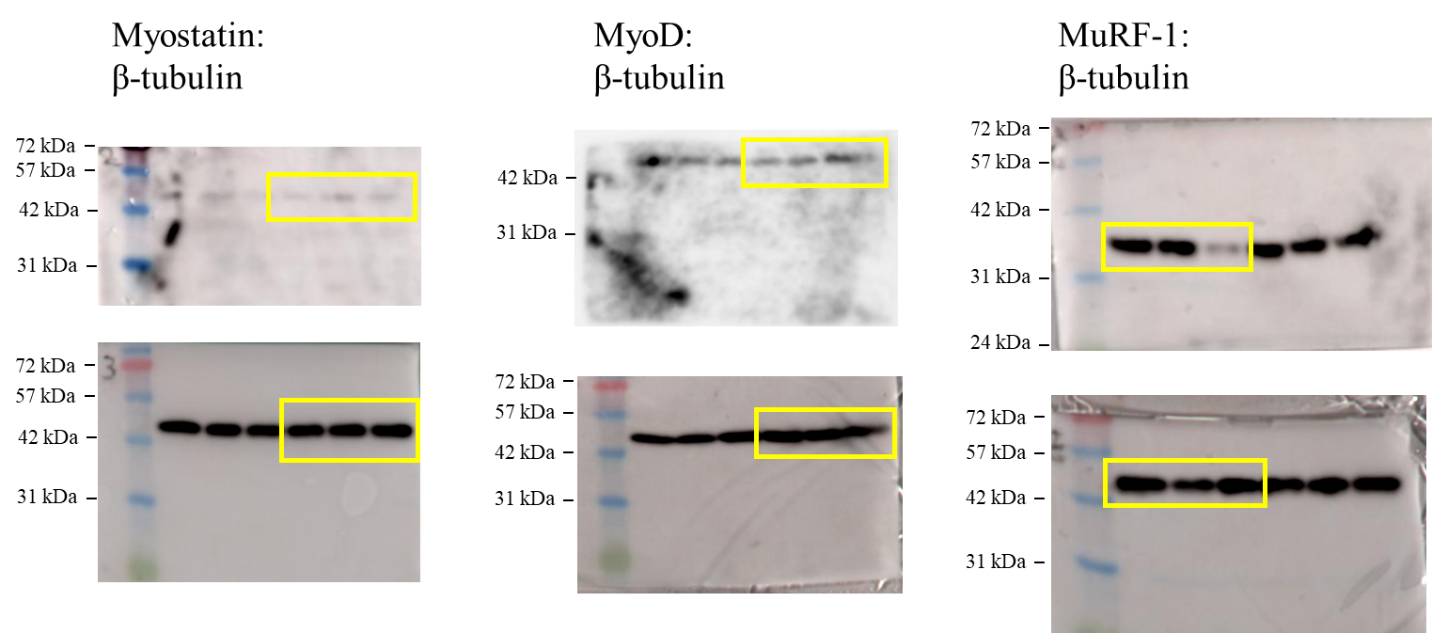


Figure S3. The original image of western blot analysis

This is the original image of western blot analysis used for Figure 4.
